# Radiology and the medical student: do increased hours of teaching translate to more radiologists?

**DOI:** 10.1259/bjro.20230029

**Published:** 2023-06-13

**Authors:** Aisha Shaheen Hameed, Aneesa K Hameed

**Affiliations:** 1 ST2 Radiology Registrar, Canterbury Hospital, Canterbury, United Kingdom; 2 King’s College London, London, United Kingdom

To the Editor,

Thank you for publishing the research paper conducted by Chew and colleagues titled “Radiology and the medical student: do increased hours of teaching translate to more radiologists?”.^1^ We read this with great interest and would like to delve into this important topic further.

The authors concluded that increased exposure to radiology teaching did not translate in increasing the radiology workforce. They acquired the figures by reviewing the General Medical Council specialist register for Clinical Radiologists and with reviewing the primary medical qualification being awarded in any of the Scottish Medical Schools. The hours of teaching was via reviewing the timetabled teaching in the curriculum for 2015 alone.

They identified that despite the workforce shortfall, the application process for training is highly competitive, with applications doubling over the decade with no translated increase in training numbers. Since the paper has been published, there has been further increase in the training numbers available, yet radiology remains competitive.^2^ As mentioned by the authors, maybe the solution to this issue is primarily a political one with increased training numbers becoming available. We believe that asking new recruits to radiology, will provide invaluable knowledge into when and why they decided to pursue a career in radiology. We believe questionnaires to this group, would clearly answer what encourages trainees to join radiology.

We found it interesting to see the amount of timetabled teaching that was available for the different universities. Glasgow, as expected with a problem-based learning curriculum, had the shortest amount of timetabled teaching. However, it had the most amount of hours for radiology specific teaching. We wondered why this had occurred and possibly whether they had changed their curriculum recently. Nevertheless, the raw figures are staggering, and demonstrate that at most radiology teaching was only 0.8% of the total teaching. This is a cause for concern, as there is an increase in demand for imaging, which has further increased due to the COVID-19 pandemic as reflected by the Royal College of Radiologists Workforce report. They concluded that the current consultant radiologist workforce shortfall was at 29%.^3^

The authors acknowledged a limitation was from excluding informal teaching, which from our experience, was the primary way that we had radiology teaching. This may have resulted in lower than expected total amount of teaching. Furthermore, it is unclear what type of teaching was included and whether this was specific to a certain module in the curriculum. From both of our personal experiences, certain rotations had more radiology-specific teaching. However, the trend was clear, that the formal teaching from radiologists did not translate into more radiologists. This suggests that a solution to the workforce is not related to radiology-specific teaching, but maybe due to a lack of training numbers, as it continues to be oversubscribed.^2^

We are at different stages in our careers. As a new recruit into the radiology, I do believe that with greater exposure in medical school to what a radiologists job involves, I would have pursued a career in radiology earlier. I embarked in the decision to apply to radiology 6 years after graduating from medical school after completing core medical training first. This feeling is mirrored by the work conducted by Oliver and colleagues, that final year medical students were unaware of the tasks of a radiologist.^4^ This is addressed at medical schools now, with radiology societies. However, we believe we need to go further, as one would not join a society when they may have a negative, yet historical perception of the career. This has been further shown by Xu and colleagues that longer time spent in the interventional radiology department and attending further educational experiences, was associated with a strong motivation to pursuing a career in interventional radiology.^5^

In conclusion, Chew and colleagues have provided an invaluable insight into the growing issue of the current shortfall in radiology consultants that the National Health Service faces. However, most radiology trainees are commencing after their foundation years.^6^ We believe future research looking into what inspired these trainees to seek a career in radiology would be invaluable to understand how to best to overcome the historical myths of a radiology career and continue the desire to join a rewarding career.
